# SGLT2 Inhibition and cardiovascular events: why did EMPA-REG Outcomes surprise and what were the likely mechanisms?

**DOI:** 10.1007/s00125-016-3956-x

**Published:** 2016-04-25

**Authors:** Naveed Sattar, James McLaren, Søren L. Kristensen, David Preiss, John J. McMurray

**Affiliations:** BHF Glasgow Cardiovascular Research Centre, Institute of Cardiovascular and Medical Sciences, University of Glasgow, 126 University Avenue, Glasgow, G12 8TA UK; Department of Cardiology, Gentofte Hospital, Copenhagen, Denmark; Clinical Trial Service Unit and Epidemiological Studies Unit, Nuffield Department of Population Health, University of Oxford, Oxford, UK

**Keywords:** Blood pressure, Cardiovascular mortality, Empagliflozin, Haemodynamic, Heart failure, Renal dysfunction, Review, Sodium-glucose linked transporter-2, Type 2 diabetes

## Abstract

While the modest reduction in the primary composite outcome of myocardial infarction, stroke or cardiovascular death in the EMPA-REG Outcomes trial was welcome, the 30–40% reductions in heart failure hospitalisation (HFH) and cardiovascular and all-cause deaths in patients treated with empagliflozin were highly impressive and unexpected. In this review, we discuss briefly why cardiovascular endpoint trials for new diabetes agents are required and describe the results of the first four such trials to have reported, as a precursor to understanding why the EMPA-REG Outcomes results came as a surprise. Thereafter, we discuss potential mechanisms that could explain the EMPA-REG Outcomes results, concentrating on non-atherothrombotic effects. We suggest that the main driver of benefit may derive from the specific effects of sodium-glucose linked transporter-2 (SGLT2) inhibition on renal sodium and glucose handling, leading to both diuresis and improvements in diabetes-related maladaptive renal arteriolar responses. These haemodynamic and renal effects are likely to be beneficial in patients with clinical or subclinical cardiac dysfunction. The net result of these processes, we argue, is an improvement in cardiac systolic and diastolic function and, thereby, a lower risk of HFH and sudden cardiac death. We also discuss whether other drugs in this class are likely to show similar cardiovascular benefits. Finally, areas for future research are suggested to better understand the relevant mechanisms and to identify other groups who may benefit from SGLT2 inhibitor therapy.

## Introduction: cardiovascular endpoint trials for new diabetes agents

Recent epidemiological studies indicate that diabetes approximately doubles cardiovascular risk [1]. It also increases mortality risk from non-cardiovascular causes, including premature death from cancers. Fortunately, cardiovascular and mortality risks have declined over recent decades, due to reductions in atherogenic lipids, blood pressure and smoking rates, and better glycaemia management. Greater absolute cardiovascular benefits appear to accrue more from reducing LDL-cholesterol and blood pressure than from intensively targeting glycaemia [2].

Notably, the results of recent trials comparing intensive glucose control with standard control led to some concern that aggressive lowering of glucose levels in some individuals may increase short-term mortality, as seen in the Action to Control Cardiovascular Risk in Diabetes (ACCORD) study [3], even though meta-analyses of such trials did confirm a modest reduction in coronary events. Furthermore, initial concerns (now largely refuted [4]) about the cardiovascular safety of rosiglitazone led the US Food and Drug Administration to mandate that all new glucose-lowering agents must be tested for cardiovascular safety in post-marketing endpoint trials. Consequently, a multitude of such trials are ongoing. It should be borne in mind that these trials are primarily designed to assess cardiovascular safety and, as such, are typically powered to demonstrate non-inferiority (defined as upper boundary of the 95% CI of the HR < 1.3) with superiority included as a subsequent statistical test (defined as upper boundary of the HR < 1.0). Five major trials have recently been published. Four of these trials investigated drugs that act on the incretin pathway (three dipeptidyl peptidase-4 [DPP-4] inhibitor trials and one glucagon-like peptide-1 [GLP-1] receptor agonist trial), with the most recent being the first sodium-glucose linked transporter-2 (SGLT2) inhibitor trial. This short review summarises the results of the first four trials as an important precursor to understanding why the results of the EMPA-REG Outcomes trial took clinicians by surprise [5]. The review then examines the possible mechanisms responsible for the benefit observed in EMPA-REG Outcomes.

## What did the trials with DPP-4 inhibitors and GLP-1 receptor agonists show?

The four trials were not designed as glucose-lowering trials per se since background glucose-lowering medications (other than incretin therapy) were generally allowed to be changed freely in both arms, in line with usual care. DPP-4 inhibitors are glucose-lowering agents that neither increase weight nor cause hypoglycaemia and that have negligible effects on lipids or blood pressure (Table 1). That noted, meta-analyses of shorter-term DPP-4 inhibitor studies seemed to suggest that cardiovascular risk might be significantly lowered in line with as yet unknown ‘pleiotropic’ effects.

**Table 1:** The broad effects on commonly considered risk factors of differing classes of new diabetes agents

Risk factor	DPP-4 inhibitors	GLP-1 receptor agonists	SGLT2 inhibitors
Hypoglycaemia risk	Low	Low	Low
Weight	Neutral	Reduced	Reduced
Blood pressure	Neutral	Lower	Lower
Lipids	Neutral	HDL-cholesterol ↑ / triacylglycerols ↓	Mixed (LDL-cholesterol ↑ / HDL-cholesterol ↑ / triacylglycerols ↓)
Other miscellaneous effects of potential relevance	Increase in heart failure in some trials	Heart rate ↑ variably among different GLP-1 receptor agonists	Genital infections

The results of the first two DPP-4 inhibitor trials (Saxagliptin Assessment of Vascular Outcomes Recorded in Patients with Diabetes Mellitus [SAVOR]–Thrombolysis in Myocardial Infarction [TIMI] 53 [6] and Examination of Cardiovascular Outcomes with Alogliptin versus Standard of Care [EXAMINE] [7], testing saxagliptin and alogliptin, respectively, as add-on therapies) were presented at the European Society of Cardiology annual meeting in September 2014. These two trials were conducted largely in high-risk patients (secondary prevention or acute coronary syndrome patients) to allow rapid accumulation of cardiovascular events and, consequently, were short in duration (Table 2).

**Table 2:** A simple overview of the five major diabetes trials of newer agents reported to date

Therapy	Trial	*N*	Population	Follow-up duration	HbA_1c_ difference during follow-up (%)	Primary outcome
Saxagliptin (DPP-4 inhibitor)	SAVOR−TIMI 53	16,492	+CVD (80%) or –CVD at high risk (20%)	24 months	0.2–0.3%	CVD death, NF MI or stroke
Alogliptin (DPP-4 inhibitor)	EXAMINE	5,380	MI or UA within last 15–90 days	18 months	0.36%	CVD death, NF MI or stroke
Sitagliptin (DPP-4 inhibitor)	TECOS	14,671	+CVD	36 months	0.3%	CVD death, NF MI or stroke, hospitalisation for UA
Lixisenatide (GLP-1R agonist)	ELIXA	6,068	MI or UA within last 180 days	25 months	0.27%	CVD death, NF MI or stroke, hospitalisation for UA
Empagliflozin (SGLT2 inhibitor)	EMPA-REG Outcomes	7,020	+CVD	37 months	0.3–0.5%	CVD death, NF MI or stroke

CVD, cardiovascular disease; ELIXA, Evaluation of Lixisenatide in Acute Coronary Syndrome; MI, myocardial infarction, NF, non-fatal; UA, unstable angina

The trials reported modest HbA_1c_ differences between treatment arms and demonstrated non-inferiority for cardiovascular events (SAVOR–TIMI 53, HR 1.00 [95% CI 0.89, 1.12]; EXAMINE, HR 0.96 [upper boundary of one-sided repeated CI 1.16]). One concern was a slight but significant increase in risk of heart failure hospitalisation (HFH) seen with saxagliptin in the SAVOR−TIMI study. EXAMINE also showed a higher risk of HFH [7] and, although not significant, the pooled results of SAVOR−TIMI and EXAMINE did raise a suspicion that this class of drugs may be associated with an increase in the risk of heart failure. This point has since been heavily debated and further studies or trials with these agents are being planned specifically to examine effects on cardiac structure and function.

The third DPP-4 inhibitor trial was the Trial Evaluating Cardiovascular Outcomes with Sitagliptin (TECOS), which tested sitagliptin in patients with established cardiovascular disease [8]. The duration of this trial was somewhat longer and the difference in HbA_1c_ between treatment arms was again small (Table 2). The results showed neutrality with respect to cardiovascular outcomes (HR 0.98 [95% CI 0.88, 1.09]) and there was no evidence for an increase in HFH. The fourth trial published in this series of new diabetes agents tested lixisenatide, a short acting GLP-1 receptor agonist, in patients with recent acute coronary syndrome [9]. Given that GLP-1 receptor agonists lower not only glucose but also weight and blood pressure [10], it was hoped that the results would demonstrate cardiovascular benefit. Again, the trial demonstrated non-inferiority for cardiovascular events (HR 1.02 [95% CI 0.89, 1.17]) with no suggestion of benefit. Similarly, HFH and mortality were unchanged.

## The value of cardiovascular endpoint trials for new glucose-lowering agents

While the availability of new glucose-lowering agents that have been shown to be safe in robust trials has substantially expanded the treatment options available, it appears that demonstrating not only non-inferiority but actual clinical benefit, in the context of falling cardiovascular event rates, would require even larger studies with longer follow-up than have yet been considered. Although assessing safety is critically important, trials of agents with modest effects on HbA_1c_ which are conducted in specific high-risk populations (e.g. those with recent acute coronary syndrome, arguably limiting the ability to impact upon major cardiovascular events within the first year) over relatively short durations seem unlikely to have the capacity to show benefit, even if the agent in question is superior to standard practice. It is important to stress that the results of Liraglutide Effect and Action in Diabetes: Evaluation of Cardiovascular Outcome Results (LEADER; testing liraglutide; ClinicalTrial.gov registration no. NCT01179048 [11]) and Exenatide Study of Cardiovascular Event Lowering Trial (EXSCEL; testing once-weekly exenatide; ClinicalTrial.gov registration no. NCT01144338) are eagerly awaited since they involve GLP-1 receptor agonists that have a longer duration of action than lixisenatide and have stronger glucose- and weight-lowering effects [12]. Hence, the effect of GLP-1 receptor agonists on cardiovascular outcomes should remain an open question for now.

## SGLT2 Inhibitor class: EMPA-REG Outcomes

The aforementioned neutral results of trials might explain why, when EMPA-REG Outcomes data were reported at the EASD annual meeting in Stockholm in 2015, the audience were highly surprised with the reductions in cardiovascular mortality, all-cause mortality and HFH for empagliflozin vs placebo. Could these results have been predicted based on what we knew about the mode of action? At the level of commonly considered risk factor changes, the answer is no—see Table 1. While SGLT2 inhibitors lower not only glucose but also weight and blood pressure, their effects on lipids are mixed with parallel rises in LDL-cholesterol and HDL-cholesterol [13]. Such risk factor patterns therefore made it impossible to predict the net effect of SGLT2 inhibitors on cardiovascular outcomes.

EMPA-REG Outcomes tested empagliflozin at two doses (10 and 25 mg daily) vs placebo in patients with existing cardiovascular disease over about 3 years. In keeping with what is known about the drug’s efficacy, active treatment compared with placebo led to reductions in HbA_1c_ (by ~0.3–0.5%), weight (by ~2 kg) and systolic blood pressure (by ~3 mmHg) without any compensatory increase in heart rate [5]. There were also slight rises in LDL-cholesterol and HDL-cholesterol levels as expected. The primary outcome of this trial was the ‘MACE’ (major adverse cardiac events) composite of death from cardiovascular causes, non-fatal myocardial infarction or non-fatal stroke, recommended in the US Food and Drug Administration’s guidance on evaluating new glucose-lowering agents and used in most of the other trials discussed earlier. While the primary outcome was reduced by empagliflozin (HR 0.86 [95 CI 0.74, 0.99]), myocardial infarction was not significantly reduced (HR 0.87 [95% CI 0.70, 1.09]), although it was directionally concordant, and stroke was non-significantly increased (HR 1.18 [95% CI 0.89, 1.56]) despite the fall in blood pressure [5]. The pattern of results from other pre-specified outcomes revealed something rather different. Empagliflozin significantly lowered death from cardiovascular causes (by 38%), HFH (by 35%) and death from any cause (by 32%) [5]. Although one must be cautious in interpreting these results on the basis that they were secondary outcomes and while further trial evidence is crucial, such a substantial reduction in all-cause death in particular gives confidence in their validity. It was the reduction in cardiovascular death that primarily drove the reduction in the primary endpoint. These benefits on mortality and HFH emerged very rapidly, almost immediately. Reassuringly, other than the expected increase in genital infections with empagliflozin (6.4% vs 1.8%), there was no increase in rates of diabetic ketoacidosis, fractures or hypoglycaemia [5]. There is now considerable interest in determining the potential underlying mechanisms for the EMPA-REG Outcomes findings.

## What potential mechanisms could explain the benefits?

A non-atherothrombotic explanation for the benefits is suggested by two important aspects from the EMPA-REG Outcomes trial: first, by substantial reductions in HFH and cardiovascular mortality rather than any clear effect on myocardial infarction or stroke risk; second, by the very rapid emergence of benefit as shown by the separation of Cumulative Incidence curves for cardiovascular mortality and HFH following randomisation [5]. To better understand what may have happened, we need to revisit the pathophysiology of heart failure.

Heart failure is defined by the inability of the heart to deliver sufficient oxygen to peripheral organs and clinically as a syndrome defined by the presence of symptoms such as ankle swelling, dyspnoea and fatigue and signs such as elevated venous jugular pressure and pulmonary crackles. It can arise as a result of almost any abnormality of the structure, mechanical function or electrical activity of the heart and is the common, end-stage, manifestation of many cardiovascular diseases including myocardial infarction. Once heart failure ensues, reduced systolic (contraction) and/or diastolic (relaxation) function may lead to sodium and fluid retention, due to renal haemodynamic changes and as a result of activation of the renin—angiotensin—aldosterone system, sympathetic nervous system (SNS) and other neurohumoral systems. SNS activation occurs in an attempt to maintain cardiac output, through increased contractility (stroke volume) and heart rate, and redistribute blood flow (but at the expense of enhanced systemic vasoconstriction). Heart failure is characterised by a trajectory of deteriorating cardiac output and declining renal function leading to fluid retention, peripheral oedema and pulmonary congestion, which if severe may lead to hospitalisation and treatment with intravenous diuretic. Of course there is a strong link between heart failure and sudden death: dilated and hypertrophied cardiac chambers fail and fibrillate, so patients may die from either pump failure or sudden arrhythmia.

At baseline, 10% of EMPA-REG Outcomes participants were clinically recorded as having heart failure, and it is likely that a further proportion of this high cardiovascular risk population had subclinical cardiac dysfunction. The results of the trial suggest that empagliflozin somehow interrupts one or more of the mechanisms involved in the progression of cardiac dysfunction mentioned above. We postulate that the relevant effect or effects of empagliflozin may involve the mechanisms included in the text box below.

**Table:** 

**Possible mechanisms for effects of empagliflozin on reduced risk of heart failure**
• Diuresis leading to reduced extracellular fluid volume (reflected in a rise in haematocrit) and cardiac pre-load, an action similar to that obtained with conventional diuretics
• One or more peripheral vascular actions leading to reduced cardiac pre- and afterload and lower systolic blood pressure, and thereby providing an important alleviation of cardiac stress
• Improved cardiac metabolism, enhancing diastolic and systolic function. Of interest, SGLT1 rather than SGLT2 receptors have been found in cardiac tissue so direct effects of empagliflozin on cardiac function appear unlikely
**Less predictable mechanisms that could contribute to a reduced risk of heart failure**
• Suppression of adverse neurohumoral systems, although the lack of increase in heart rate suggests no further SNS activation
• Reduction in myocardial ischaemia, unrecognised/silent myocardial infarction or other causes of cardiomyocyte necrosis
• Reduction in pathological growth (hypertrophy and fibrosis)
• Reduction in arrhythmias
• Greater use of other agents in placebo arm that cause weight gain, or directly increase fluid load (thiazolidinediones)

## Relevance of blood pressure and renal effects

Whether the blood pressure-lowering effects of empagliflozin alone can explain the observed benefit is worthy of consideration since reducing blood pressure (even by small amounts) can influence heart failure risks, although not necessarily as rapidly as seen in EMPA-REG Outcomes. Empagliflozin also induces a considerable diuresis, with early loss of urinary glucose in particular and subsequently of sodium as reflected by a sizeable increase in the haematocrit (~4%) compared with placebo, and certainly adequate dosage of diuretics can rapidly reduce heart failure risks, or risks for HFH [14]. Notably, in the Antihypertensive and Lipid-Lowering Treatment to Prevent Heart Attack Trial (ALLHAT), in which just over one-third of patients had type 2 diabetes, use of the thiazide diuretic chlorthalidone was associated with a 28% (95% CI 20, 34) lower risk of heart failure compared with amlodipine despite only a 0.8 mmHg difference in achieved systolic blood pressure [15]. That noted, there were no differences in other vascular endpoints.

**Fig. 1:**
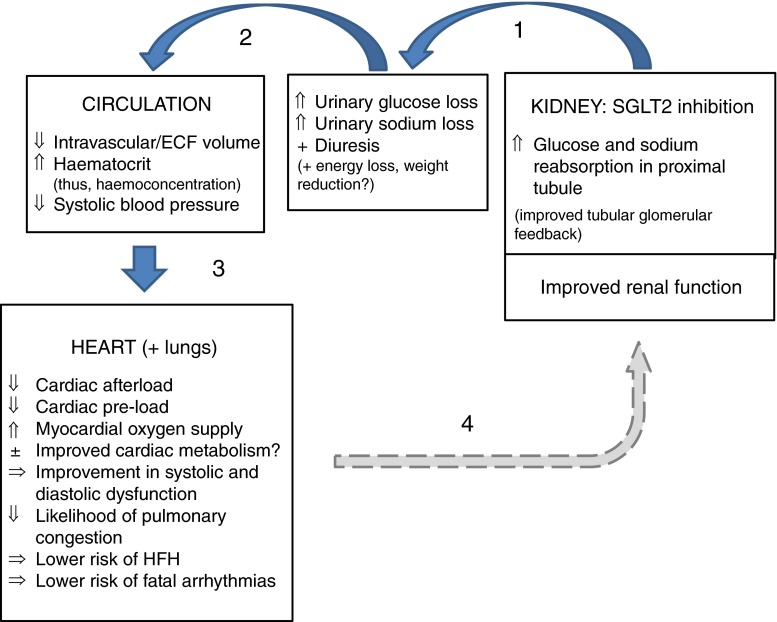
Potential pathway linking empagliflozin (and possibly other SGLT2 inhibitors) with lower risks for HFH (and, linked to this, death due to cardiovascular disease). By increasing fluid losses via urinary glucose and sodium losses (**1**), intravascular volumes and systolic blood pressure are reduced and there is a significant rise in haematocrit (**2**). These latter effects may also be, to a small extent, assisted by weight loss. These changes in turn lessen cardiac stressors (pre- and afterload) and may also help improve myocardial oxygen supply (**3**). The net result is a likely improvement in cardiac systolic and diastolic function, lessening chances of pulmonary congestion, thus lowering risks of HFH and fatal arrhythmias. These cardiac function benefits will, in turn, feed back to improve renal blood flow and function (**4**). In this way, the cardio-renal axis is improved at a number of levels with SGLT2 inhibitor therapy

We consider that the cardiovascular benefit of empagliflozin is related to the manner in which it induces diuresis (both glucose and sodium losses), notably with a reduction in the progression to renal failure and with a slowing in the deterioration of renal function. Type 2 diabetes is typified by upregulated SGLT2 tubular transporters and increased tubular glucose reabsorption, along with sodium reabsorption in the proximal renal tubules, leading to decreased sodium delivery to the macula densa, vasodilatation of the afferent arteriole with vasoconstriction of the efferent arteriole and resultant intraglomerular hypertension. The combination of the diuretic effect and increased sodium delivery to the macula densa [16], thereby helping to address the maladaptive arteriolar responses, may explain the finding of both cardiac and renal benefit of SGLT2 inhibition. In essence, improving renal sodium and glucose handling, with subsequent reductions in fluid burden especially in individuals with or susceptible to cardiac dysfunction, may have been the key driver underlying the benefits seen in EMPA-REG Outcomes (Fig. 1). There is a need for detailed mechanistic studies incorporating state of the art imagining techniques to better determine the full cardiovascular actions of empagliflozin and other SGLT2 inhibitors in patients with diabetes. Such studies should involve a range of diabetes patients with and without cardiovascular disease and with and without existing heart failure. In addition, major trials will be required to establish whether SGLT2 inhibitor therapy, with its proposed novel diuretic-like action, might provide clinically meaningful benefit in lower-risk populations.

## Other important lessons from EMPA-REG Outcomes

There have been secular decreases in myocardial infarction and stroke rates in patients with diabetes, reflecting considerable improvements in lipid and blood pressure management over the last two decades. Indeed, in EMPA-REG Outcomes, more than 75% of patients were on statins and around 95% on antihypertensive agents at baseline, and baseline LDL-cholesterol and blood pressure results would be considered excellent in most clinical settings. However, heart failure remains an under-recognised complication of diabetes and one which carries a poor prognosis [17]. In a recent observational analysis of patients with diabetes, heart failure was found to be the second-most common type of first cardiovascular disease presentation (after peripheral vascular disease) [18]. In diabetes, the pathogenesis of heart failure is likely to be multi-factorial given the presence of risk factors including dysglycaemia, coronary heart disease, hypertension, obesity, renal dysfunction and others. It is therefore increasingly apparent that therapeutic approaches that also reduce the development and progression of heart failure (i.e. beyond atherothrombotic targeting) are crucial in patients with diabetes.

## Unanswered questions

Why stroke risk in EMPA-REG Outcomes did not decline on empagliflozin, when the risk of stroke is normally very sensitive to reductions in blood pressure, is not clear. Meta-analysis of trials suggests that stroke risks can be significantly lowered even when baseline systolic blood pressure is <140 mmHg [19] and so further investigations are clearly required. That the haemoconcentration effect of SGLT2 inhibition may have balanced out the blood pressure-lowering effect is plausible, and results from other major ongoing trials will yield valuable data.

## Will other SGLT2 inhibitors give the same results as EMPA-REG Outcomes?

If we assume that the explanations for the benefit of empagliflozin revolve around its effects principally on diuresis (driven by promoting both glucose and sodium loss) and via blood pressure reductions, then other SGLT2 inhibitors should yield directionally similar outcomes. That noted, available SGLT2 inhibitors have differential specificities for SGLT2 and SGLT1 [20], the relevance of which is not clear for the moment. Fortunately, we will not have to wait too long before know the results of the ongoing trials for canagliflozin (Canagliflozin Cardiovascular Safety Assessment [CANVAS] due to be completed in 2017; ClinicalTrial.gov registration no. NCT01032629) [21] and dapagliflozin (Dapagliflozin Effect on CardiovascuLAR Events [DECLARE]-TIMI 58, due to be completed in 2019; ClinicalTrial.gov registration no. NCT01730534 [www.timi.org/index.php?page=declare-timi-58, accessed 1 April 2016]).

## Summary

The effects of empagliflozin on important secondary endpoints in EMPA-REG Outcomes, namely HFH, cardiovascular and all-cause death, were unexpected. The rapid emergence of these benefits points strongly towards non-atherothrombotic mechanisms, perhaps principally haemodynamic effects. Further mechanistic studies will be needed to identify these mechanisms and further clinical trials of SGLT2 inhibitors in patients with and without heart failure, with and without cardiovascular disease and, potentially, with and without diabetes, will likely ensue. Finally, we will also shortly know whether these findings are common to other drugs in the class. It appears that a new era in diabetes–cardiovascular research has emerged.

## Abbreviations

DPP-4: Dipeptidyl peptidase-4
EXAMINE: Examination of Cardiovascular Outcomes with Alogliptin versus Standard of Care
GLP-1: Glucagon-like peptide-1
HFH: Heart failure hospitalisation
SAVOR-TIMI: Saxagliptin Assessment of Vascular Outcomes Recorded in Patients with Diabetes Mellitus–Thrombolysis in Myocardial Infarction
SGLT2: Sodium-glucose linked transporter-2
SNS: Sympathetic nervous system
TECOS: Trial Evaluating Cardiovascular Outcomes with Sitagliptin
